# Incorporation of Arginine to Commercial Orthodontic Light-Cured Resin Cements—Physical, Adhesive, and Antibacterial Properties

**DOI:** 10.3390/ma14164391

**Published:** 2021-08-05

**Authors:** Saulo Geraldeli, Lucas de Almeida Maia Carvalho, Isaac Jordão de Souza Araújo, Maurício Bottene Guarda, Marcelle M. Nascimento, Marcus Vinícius Loureiro Bertolo, Paolo Túlio Di Nizo, Mário Alexandre Coelho Sinhoreti, V. Wallace McCarlie

**Affiliations:** 1 Department of General Dentistry, Division of Biomedical Materials, School of Dental Medicine, East Carolina University, Greenville, NC 27834-4354, USA; 2 Department of Restorative Dentistry, Piracicaba Dental School, University of Campinas, Campinas 13414-903, SP, Brazil; lucas.almeidamaiacarvalho@gmail.com (L.d.A.M.C.); isaacjsouzaa@gmail.com (I.J.d.S.A.); Mauricio_Guarda@hotmail.com (M.B.G.); marcusbertolo@hotmail.com (M.V.L.B.); paolodinizo@gmail.com (P.T.D.N.); sinhoreti@fop.unicamp.br (M.A.C.S.); 3 Department of Restorative Dental Sciences, College of Dentistry, University of Florida, Gainesville, FL 32610-0412, USA; mnascimento@dental.ufl.edu; 4 Department of Pediatric Dentistry and Orthodontics, Division of Orthodontics and Dentofacial Orthopedics, School of Dental Medicine, East Carolina University, Greenville, NC 27834-4354, USA; mccarlievw@ecu.edu

**Keywords:** adhesive systems, orthodontic resin cements, brackets, arginine

## Abstract

(1) Background: The amino acid arginine is now receiving great attention due to its potential anti-caries benefits. The purpose of this in vitro study was to evaluate the shear bond strength (SBS), ultimate tensile strength (UTS), and antimicrobial potential (CFU) of two arginine-containing orthodontic resin cements. (2) Methods: Forty bovine incisors were separated into four groups (n = 10): Orthocem, Orthocem + arginine (2.5 wt%), Transbond XT, and Transbond XT + arginine (2.5 wt%). The brackets were fixed to the flat surface of the enamel, and after 24 h the SBS was evaluated using the universal testing machine (Instron). For the UTS test, hourglass samples (n = 10) were made and tested in a mini-testing machine (OM-100, Odeme). For the antibacterial test (colony forming unit-CFU), six cement discs from each group were made and exposed to *Streptococcus mutans* UA159 biofilm for 7 days. The microbiological experiment was performed by serial and triplicate dilutions. The data from each test were statistically analyzed using a two-way ANOVA, followed by Tukey’s test (α = 0.05). (3) Results: The enamel SBS mean values of Transbond XT were statistically higher than those of Orthocem, both with and without arginine (*p* = 0.02033). There was no significant difference in the SBS mean values between the orthodontic resin cements, either with or without arginine (*p* = 0.29869). The UTS of the Transbond XT was statistically higher than the Orthocem, but the addition of arginine at 2.5 wt% did not influence the UTS for either resin cement. The Orthocem + arginine orthodontic resin cement was able to significantly reduce *S. mutans* growth, but no difference was observed for the Transbond XT (*p* = 0.03439). (4) Conclusion: The incorporation of arginine to commercial orthodontic resin cements may be an efficient preventive strategy to reduce bacterial growth without compromising their adhesive and mechanical properties.

## 1. Introduction

Dental malocclusions are still prevalent in the world’s population, and one of the recommended treatments relies on the bonding of orthodontic brackets to enamel to correct tooth positioning [1,2,3,4]. The drawback with this fixing orthodontic strategy, however, is that it favors biofilm accumulation, and limits oral self-cleaning mechanisms and the patient’s ability for effective removal. Subsequently, this protected microbiota increases its activity and acid production, leading to the demineralization of the enamel structure around the brackets, which conduces to the first clinical sign of tooth decay (i.e., the white spot lesion (WSL) [5,6]), and it is considered a common adverse effect in fixed orthodontic treatment [7]. As a matter of fact, the evidence shows that this type of caries lesion is prevalent in more than 60% of patients undergoing orthodontic treatment [8,9].

Strategies to reduce the incidence of WSL during and after fixed orthodontic treatment have been recommended and tested. Among them, the use of chemical therapy with mouthwashes and chairside fluoride varnish application are common [10,11]. Despite the complementary therapies, the presence of carious lesions persists. Therefore, new strategies for the control of WSL associated with orthodontic treatment are highly desired.

Arginine enters the mouth through dietary components (normal intake of 5 g/day in adults) [12] but is also naturally produced by the human body via protein turnover and de novo arginine synthesis from citrulline. In supragingival biofilms, arginine is metabolized mainly by the arginine deiminase system (ADS) of certain bacteria to produce citrulline, ornithine, CO_2_, ATP, and ammonia. Ammonia production via ADS has a positive impact on the pH homeostasis of supragingival biofilms [13,14,15,16,17,18]. Toothpastes or mints containing arginine were demonstrated to be highly effective at inhibiting caries initiation and progression [19,20]. Of particular importance, our research group showed that the incorporation of arginine into dental adhesives may be a cost-effective strategy to prevent secondary caries around the margins of resin composite restorations [21].

An ideal fixative material should have adequate adhesive strength to resist movements during orthodontic and chewing forces. In addition, it should reduce the decalcification indexes on enamel around the bracket [22]. Glass ionomer cement (GIC) was developed in 1972 by Wilson and Kent, and has the ability to release ions, like fluorine and aluminum, which makes this material anticariogenic [23]. On the other hand, GIC has poor cohesive resistance, which implies inferior mechanical adhesive properties to the teeth when compared to resin cement [24]. In this sense, the use of resin cements is more reliable to bond orthodontic brackets to the teeth.

Regarding the antibiofilm or anticariogenic potential of composites materials, there is no evidence supporting its superiority if compared to other dental conventional materials. Nevertheless, quaternary ammonium monomers have already been added to the composites and bonding systems [25,26,27] to reduce, or even inhibit, bacterial growth. Besides ammonium quaternary, chlorhexidine and other antimicrobial agents were also used to develop anticariogenic resin composites [28]. Based on the need for antimicrobial activity in resin composites, it is necessary to test novel materials presenting compounds with verified efficacy against oral biofilms. In this sense, studies show that the use of arginine in candies and dentifrices was highly effective in inhibiting the initiation and progression of dental caries [19,20]. Arginine is an amino acid that can be found in several foods, in addition to being produced by the human body. It is secreted by saliva in a free form or as salivary peptides. Of particular importance, a recent study by Geraldeli et al. [21] showed that the addition of arginine in dental adhesives can promote anticariogenic activity without damaging the mechanical and adhesive properties. In addition, arginine metabolism contributes to the maintenance of pH in the oral environment and helps to regulate the oral microbiota [29,30,31].

Therefore, combining the need to develop antibacterial resin cements for brackets fixation and the satisfactory results of arginine in previous studies, it was decided to carry out this study. Thus, the aim was to evaluate the influence of arginine addition on commercial orthodontic light cure resin cements in terms of ultimate tensile strength, shear bond strength, and its antibacterial potential. The two-null hypotheses tested were: (1) The addition of arginine to commercially orthodontic luting resin cement would not influence in the material’s ultimate tensile strength and the enamel shear bond strength; (2) The addition of arginine would not reduce the growth of *Streptococcus mutans (S. mutans)*.

## 2. Materials and Methods

### 2.1. Orthodontic Resin Cement Manipulation

The commercial orthodontic resin cements used in this study were Orthocem (FGM, Joinville, SC, Brazil) and Transbond XT (3M ESPE, St. Paul, MN, USA). Under orange light, the total volume of each syringe material was weighted (DV215CD, Ohaus Corporation, Pine Brook, NJ, USA) and then stored in brown recipients to prevent exposure to light. Then, 2.5 wt% of arginine (Sigma-Aldrich Inc., St. Louis, MO, USA) was added to each orthodontic resin cement and mechanically mixed in a centrifugal mixing device (DAC 150 Speed Mixer, Flacktek, Landrum, SC, USA) at 3000 rpm for 1 min, keeping the temperature below 37 °C.

Dental enamel surface preparation for evaluation of shear bond strength.

Forty bovine incisors, freshly extracted, cleaned, and stored in distilled water at 4 °C, were used (Angelelli slaughterhouse, Piracicaba, Brazil). After storage, the teeth were immersed up to 1 mm below the CEJ in PVC tubes (25 mm ø by 30 mm in length) containing self-curing acrylic resin (VIPI, Pirassununga, SP, Brazil). A 90° angle positioner was used on the buccal tooth surface at the top of the PVC tube to keep the buccal surfaces of the teeth perpendicular to the base of the tube. Then, the self-curing acrylic resin was inserted into the PVC tubes to fix the teeth. After polymerization, the specimens were again stored in distilled water and refrigerated.

Before the bonding procedures, prophylaxis with pumice paste and a rubber cup were performed on the buccal surfaces of the teeth for 30 s, followed by water rinsing and air drying. The buccal enamel was conditioned with 35% phosphoric acid gel (Ultra-Etch, Ultradent, South Jordan, UT, USA) for 30 s, then washed and dried for 30 s, as per the manufacturer’s instructions.

Table 1 describes the application protocol for each group. The brackets (Light, Prescription Roth, Morelli Orthodontics, Sorocaba, SP, Brazil) were compatible with the teeth and the photocuring procedures were performed using a light curing unit Bluephase G2 (Ivoclar Vivadent, Schaan, Liechtenstein) with 1200 mW/cm^2^ of irradiance.

### 2.2. Shear Bond Strength (SBS) Test

The SBS test was carried out in a universal testing machine (Instron Corporation, model 4411, Canton, OH, USA). The interface bracket-enamel of each specimen was positioned parallel to the edge of a knife-edged rod. The SBS tests were made at a crosshead speed of 1 mm/min using a 50 N load cell. The results were recorded by specific software and the load, recorded in N (Newtons), was converted into MPa (MegaPascal), dividing the load by the bracket area (7.29 mm^2^).

### 2.3. Adhesive Remnant Index (ARI) Test

After de-bonding, the enamel surfaces were evaluated in a stereomicroscope (25× of magnification), and the orthodontic resin cement remaining recorded using the ARI. The criteria for ARI scoring were: 0, no resin cement on the tooth surface; 1, less than 50% of resin cement on the tooth surface; 2, more than 50% of resin cement on the tooth surface; and 3, all resin cement remained on the tooth surface.

### 2.4. Ultimate Tensile Strength (UTS) Test

Hourglass shaped specimens (10 mm long × 4 mm wide × 1.5 mm thick) of each orthodontic light cure resin cement (n = 10) were prepared on a using polyvinyl siloxane molds (constriction area: 1.5 × 1.5 mm; cross-sectional area: 2.25 mm²). The photocuring procedures were performed using a light curing unit Bluephase G2 (Ivoclar Vivadent, Scahnn, Liechtenstein) with 1200 mW/cm^2^ of irradiance. The specimens were submitted to a tensile strength test in a semi-universal testing machine OM100 (Odeme Dental Research, Luzerna, SC, Brazil) at a crosshead speed of 0.75 mm/min. The UTS was calculated in MPa using the formula: UTS **=** F/A, where F is the tensile load (N) and A is the transversal cross section area (mm^2^).

### 2.5. Antibacterial Test

Eighteen discs (6 mm in diameter × 2 mm in height) of each orthodontic resin cement (with or without arginine) were made as described above. The discs were prepared under aseptic conditions in a laminar flow hood using circular rubber molds positioned on a glass plate. After the insertion of the luting cement into the mold, the samples were then covered with a polyester strip and light-cured with 32 J/cm^2^ of radiant exposure using a light curing unit (Bluephase G2, Ivoclar Vivadent). After light curing, the specimens were left under a UV light for 15 min for further decontamination.

Six specimens from each group were placed individually in a 24-well polystyrene culture plate (Kasvi) and immersed in 1.5 mL of brain heart infusion (BHI) culture medium with 1% sucrose and Xml of an inoculum of *S. mutans* UA159 strain (ATCC^®^ 700610™, Manassas, VA, USA). The plates were then incubated at 37 °C with 10% CO_2_ for 7 days. Every 24 h, the culture medium was aspirated and replaced with 1.5 mL of sterile BHI medium with 1% sucrose.

After 7 days, the resin discs were immersed in 0.9% saline solution and vortexed to detach the bacterial cells adhered to the disc. After the removal of the cells, 50 μL of the saline solution with the bacterial cells was transferred into microtubes containing 450 μL of sterile saline for serial dilution. Three drops of 25 μL of each dilution were dispensed separately into petri dishes containing a BHI agar medium. The petri dishes were stored at 37 °C with 10% CO_2_ and kept for 48 h, allowing the growth of *S. mutans* colonies. After 48 h, counting colony formation units (CFU) was performed in a stereomicroscope. The data were expressed as CFU/mL and three independent experiments were performed.

### 2.6. Scanning Electron Microscope (SEM) Analysis

One circular sample (6 mm in diameter × 2 mm in height) of each orthodontic resin luting cement was made using a rubber mold and light cured as described previously. The specimens were gold/palladium coated (Balzers-SCD 050 sputter coater, Germany) and digital images at 500× were obtained under a scanning electron microscope (JEOL-5600 LV, Japan), under a voltage acceleration of 15 KV, Z = 25 mm, WD = 20 mm, and a spot size of 27 nm.

### 2.7. Statistical Analysis

The data for the SBS, UTS, and CFU tests were submitted to the analysis of variance (two-way ANOVA) and the means were compared by Tukey’s test (α = 0.05).

## 3. Results

The mean values (MPa) and standard deviations obtained for the shear bond strength tests are presented in Table 2. The two-way ANOVA showed an interaction between the factors “resin cement” × “arginine” (*p* = 0.02033). Transbond XT showed higher bond strength than Orthocem, despite the arginine addition. The addition of arginine did not cause statistical differences in the values of shear bond strength for either orthodontic luting resin cements.

**Figure 1:**
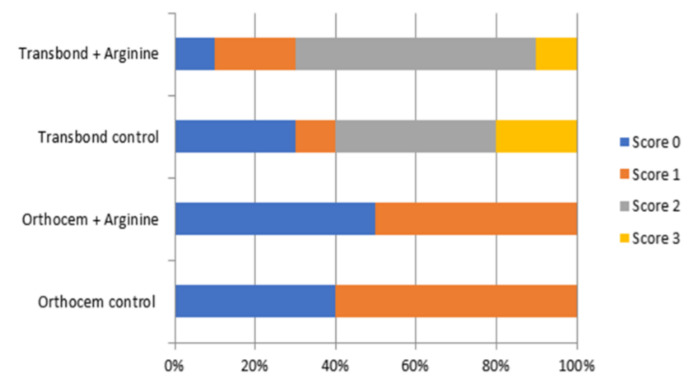
Image for the adhesive remnant index (ARI) for the material tested.

The orthodontic luting cement adhesive remnant index (ARI) results are shown in Figure 1. It can be observed that Orthocem scored zero or one in both groups. Transbond XT presented all ARI scores, with a score of two being the predominant failure mode on both groups.

Table 3 shows the UTS means. The two-way ANOVA showed a significant effect for the factor “luting resin cement” (*p* = 0.00431) and no significant difference for the factor “arginine” (*p* = 0.98326). There was no significant effect for the interaction between the factors “luting resin cement” × “arginine” (*p* = 0.08191). The addition of arginine to the Transbond and Orthocem resins did not statistically influence the UTS of these materials. Transbond XT obtained mean values statistically higher than Orthocem, both in the control and experimental groups.

Table 4 presents the mean values of *S. mutans* CFU for the of the tested orthodontic luting resin cements. The two-way ANOVA showed an interaction between the factors “resin” × “arginine” (*p* = 0.034). The capacity of Transbond XT to reduce the growth of *S. mutans* was significantly higher as compared to that of Orthocem, regardless of the addition of arginine. The addition of Orthocem significantly reduced the growth of *S. mutans*, but the addition of arginine did not reduce the antibacterial properties of Transbond XT.

**Figure 2:**
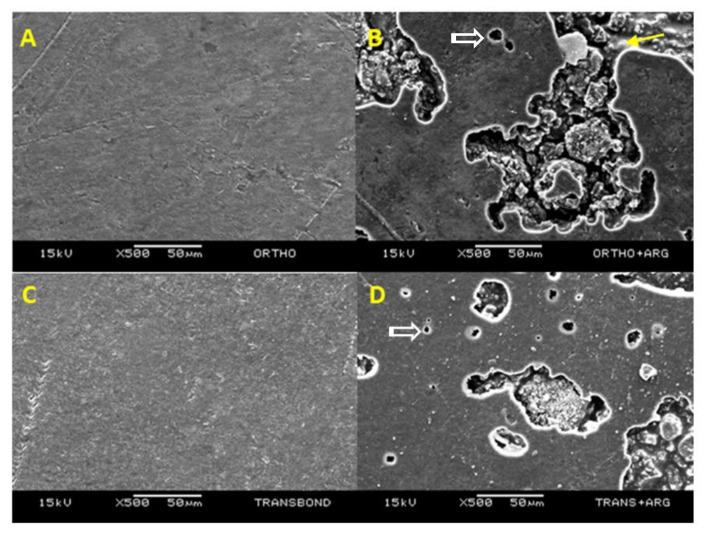
SEM images of the luting orthodontic resin cements tested. (**A**) Orthocem—The polished surface shows few discrepancies or porosities; (**B**) Orthocem + arginine —The polished surface depicts the presence of wide porosities ranging from 5 µm to 100 µm. Inside the porosity it is possible to see arginine particles covered with resin matrix (yellow arrow) as well as loose particles (white arrow); (**C**) Transbond XT—Polished surface showing minor irregularities; (**D**) Transbond XT + arginine—Polished surface showing increased number of small porosities ranging from 5 µm to 30 µm (open arrow) and fewer wider porosities. Loose arginine particles are visible (white arrow).

The image below shows SEM images illustrating the morphological aspects of the polished surface of all tested orthodontic luting resin cements (Figure 2). It can be observed that arginine particles formed clusters within the resin matrix along with a few porosities.

## 4. Discussion

The findings from this study confirmed the first study’s null hypothesis that adding 2.5 wt% arginine to the orthodontic cements would not affect the resin’s ultimate tensile strength (UTS) or the enamel shear bond strength (SBS). Comparing both commercial products, Transbond XT showed higher UTS mean values than Orthocem, regardless of the incorporation of arginine. This may be due to the higher percentage (70%) of inorganic silanized silica particles in its formulation, while Orthocem has only 48%. Moreover, the addition of 2.5 wt% arginine did not statistically influence the UTS of either orthodontic resin cements (Table 3). A similar finding was presented by Geraldeli et al. [21], in which the concentration of 7%wt of arginine to a dental adhesive system did not decrease the mechanical properties. The second study’s null hypothesis was that the incorporation of arginine would not inhibit the growth of *S. mutans*; this hypothesis was confirmed for the Orthocem but not for the Transbond XT resin cement. The addition of arginine did not statistically influence the ultimate tensile strength of either orthodontic resin cements. Geraldeli et al. [21] verified that the concentration of 7%wt of arginine in adhesive systems did not decrease the mechanical properties. In this study, 2.5% of arginine was added into the orthodontic resin cements and this low concentration did not affect the UTS (Table 3).

In the dental literature, it is reported that enamel bond strength values of 6 to 8 MPa are acceptable for clinical orthodontic success [32,33]. In this study, it was observed that all groups showed SBS means superior to the minimum required for success in clinical orthodontics (14.54 to 20.57 MPa). Despite that, Transbond XT orthodontic resin cement exhibited the highest values of shear bond strength (*p* < 0.05), even with the arginine addition. The phosphoric acid conditioning on enamel causes interprismatic dissolution and creates an irregular surface that facilitates the brackets’ retention. However, orthodontic resin cements are viscous, which makes the penetration into the etched enamel micropores difficult [34]. Therefore, after acid etching, it would be interesting to apply an unfilled resin layer on the dental surface to penetrate the micro porosities contributing to the mechanical retention of the orthodontic resin cement. In this sense, the use of an unfilled resin (Transbond XT adhesive primer), applied on the enamel prior to the Transbond XT orthodontic resin cement, may have been the reason for the higher SBS means when compared to Orthocem. This statement is also supported by Albadejo et al. [35], which found that the shear strength is more effective when prior adhesive application was done.

The ARI has been used to standardize bond failure analysis. Overall, the evaluation in this study showed a predominance of score two for all Transbond XT groups, showing that the failures occurred mainly at the interface between the bracket and the orthodontic resin cement. On the other hand, the Orthocem orthodontic resin cement presented just scores of zero and one, showing that the failures occurred at the interface between the bonding material and the tooth enamel. This corroborates the lower bond strength values obtained in the SBS test and reinforces the hypothesis that the acid-only surface treatment causes lower adhesion between orthodontic resin cement and enamel, and that the use of the adhesive primer prior to the orthodontic resin cement increases the bond strength values.

In this study, *S. mutans* was chosen because it is a gram-positive bacterium, facultative aerobic, and considered the most acidogenic species present in the biofilm [36]. The literature shows that fixed orthodontic appliances significantly increase the retention’s niches of the oral biofilm and, consequently, of *S. mutans* [37,38,39].

The second hypothesis, that the arginine addition would decrease the growth of *S. mutans*, was partially accepted since the arginine only significantly decreased the growth of *S. mutans* for the Orthocem orthodontic resin cement. 

The results of this study may be justified because when arginine is present in the mouth, it is metabolized by some oral bacteria to produce ammonia [40]. Ammonia production from the bacterial metabolism of arginine neutralizes the glycolytic acids responsible for enamel demineralization, thus reducing the risk of caries development. In fact, salivary levels of free arginine are strongly correlated with caries resistance [41]. The plaque of caries-free individuals has higher pH values compared to plaque from caries-active individuals [42,43,44], and this difference has been correlated with elevated ammonia levels in caries-free subjects [42]. Furthermore, a positive correlation between caries activity and a low arginolytic capacity of the supragingival microbial populations has been shown in adults and children [45].

Moreover, CFU results also showed that bacterial growth was statistically lower for the Transbond XT resin cement, regardless of the arginine addition. A possible factor that contributed to these findings was the presence of triphenylantimony in the Transbond XT adhesive primer, which was applied prior the Transbond XT orthodontic resin cement. In 2014, Islam et al. [46] developed a study in which two new models of triphenylantimony were synthesized, characterized, and evaluated for antibacterial activity, antileishmania, and cytotoxicity. The antimicrobial activity tests were performed quantitatively by determining the minimum inhibitory concentration (MIC). All complexes were active against gram positive bacteria. Thus, it is possible that this component present in the Transbond XT adhesive primer contributed to a higher antibacterial activity in relation to the Orthocem resin cement. However, for Orthocem orthodontic resin cement, the addition of arginine particles is an interesting strategy to reduce bacterial growth and, consequently, the appearance of carious lesions in patients whose orthodontic brackets were bonded with Orthocem.

Figure 2 shows both orthodontic resin cements with and without arginine particles. The particle agglomerates are within the porosities they create in the resin matrix. It is important to remember that these samples were not polished, as this would not reproduce a situation that occurs clinically in the cementation of orthodontic brackets, and because polishing would remove the arginine particles from the resin. However, these porosities did not decrease the UTS, as can be seen in Table 2. Nevertheless, future investigations should be carried out to improve the incorporation of arginine particles in the resinous materials, as well as to analyze the time it takes the particles to be dissolved when the resin is exposed to the oral environment, and the possibility of recharging the material with arginine. From the results found in this study and from other previous findings, including arginine into orthodontic resin luting cement seems to be a promising strategy for the control of white spot lesions and caries formation during orthodontic treatment.

## 5. Conclusions

The 2.5% arginine addition in the Transbond XT and Orthocem resin cements did not influence their ultimate tensile strength or shear bond strength and was able to decrease the growth of *S. mutans* for only the Orthocem orthodontic resin cement.

## Figures and Tables

**Table 1:** Materials used and application methods for each group/material tested.

Group/Material	Composition	Application
Orthocem control	Bis-GMA, TEGDMA, phosphated methacrylic monomers, canforquinone, tertiary amine, silicon dioxide	Cement application and light curing for 10 s on each side of the bracket.
Orthocem + Arginine (OA)	Bis-GMA, TEGDMA, phosphated methacrylic monomers, canforquinone, tertiary amine, silicon dioxide + 2.5 wt% of arginine	Cement application and light curing for 10 s on each side of the bracket.
Transbond XT control (TC)	*Primer*: Bis-GMA and TEGDMA, triphenylantimony, CQ, DMAEMA Resin: Bis-GMA, TEGDMA, BIS-EMA quartz, silicon dioxide, canforquinone, DMAEMA, DPI	Active primer application for 10 sec, cement application and light curing for 10 s on each side of the bracket.
Transbond XT + Arginine (TA)	*Primer*: Bis-GMA and TEGDMA, triphenylantimony, CQ, DMAEMA Resin: Bis-GMA, TEGDMA, BIS-EMA quartz, silicon dioxide, canforquinone, DMAEMA, DPI + 2.5 wt% of arginine	Active primer application for 10 sec, cement application and light curing for 10 s on each side of the bracket.

**Table 2:** Mean (standard deviation) values (MPa) of shear bond strength of the tested materials.

Orthodontic Cement	Control	Arginine
Transbond	20.57 (6.73) ^a,A^	17.52 (3.70) ^a,A^
Orthocem	15.17 (4.43) ^b,A^	14.54 (6.53) ^b,A^

Different small letters (^a,b^) in column and capital letters (^A,B^) in line indicate statistical difference (Tukey test, α = 0.05).

**Table 3:** Mean values and standard deviation (MPa) of UTS of the tested materials.

	Control	Arginine	Pooling Mean
Transbond XT	57.01 (10.66)	50.54 (11.68)	53.78 (11.37) ^A^
Orthocem	39.5 (8.99)	45.84 (13.92)	42.67 (11.86) ^B^
Pooling Mean	48.25 ^a^ (9.35)	48.19 ^a^	

Means followed by same small letter (^a^) in row indicate that they are not statically different and that the means followed by different capital letter (^A,B^) in column are statistically different at 5% by Tukey’s test.

**Table 4:** Mean and standard deviation (×10^−6^ CFU/mL) for *S. mutans* after incubation with the tested materials.

Material	Control	Arginine
Transbond XT	0.31 ^b,A^	0.24 ^b,A^
Orthocem	1.04 ^a,A^	0.5 ^a,B^

Different small letters (^a,b^) in column and capital letters (^A,B^) in line indicate statistical difference (Tukey’s test, α = 0.05).

## Data Availability

The study did not report any data.
